# When AI works, employees worry: the vigilance cost of AI delegation in hybrid work

**DOI:** 10.3389/fpsyg.2026.1914119

**Published:** 2026-09-24

**Authors:** Yifan Zhang, Tianyou Li, Chunming Chen

**Affiliations:** 1 College of Science and Technology Changchun, Changchun, China; 2 Ural Federal University, Yekaterinburg, Russia; 3 University of Nottingham, Nottingham, United Kingdom

**Keywords:** AI delegation dependence, AI process vigilance, artificial intelligence, boundary management, employee recovery, occupational well-being, psychological detachment, work-relatedrumination

## Abstract

Hybrid work and AI-assisted technologies have transformed not only where employees work but also how work remains psychologically active after formal working hours. This study develops the AI delegation paradox, which proposes that although AI can reduce the effort required to execute work tasks, it may simultaneously increase employees’ need to monitor, evaluate, and remain accountable for work processes that continue through AI-assisted systems. Building on this argument, the study introduces AI process vigilance (AIPV), a second-order construct capturing sustained cognitive attachment to delegated AI-assisted work through progress monitoring, output quality concern, error anticipation, and loss of control. Using a two-wave survey design with a seven-week interval, data were collected from 642 hybrid employees who used AI tools for work at least weekly. The results supported a sequential pathway in which continuous AI availability was linked to greater AI delegation dependence, delegation dependence predicted higher levels of AI process vigilance, vigilance increased work-related rumination, rumination undermined psychological detachment, and lower detachment was associated with poorer recovery outcomes. The hypothesized moderating role of boundary management preference was not supported. These findings suggest that recovery impairment may arise not only because employees continue working after hours, but also because they remain psychologically engaged with work processes that have been delegated to AI. By demonstrating that responsibility and concern often persist even when task execution is transferred to intelligent systems, the study identifies a previously overlooked recovery cost of AI-assisted work and extends current understanding of recovery, boundary management, and human–AI collaboration in contemporary work environments.

## 1 Introduction

### 1.1 AI-assisted hybrid work and the emerging recovery challenge

Hybrid work and AI-assisted technologies are becoming increasingly intertwined in contemporary knowledge work. Research on hybrid and remote work has shown that greater flexibility can reshape work design while weakening the temporal and spatial boundaries that traditionally separated work from nonwork life (Wang et al., 2021). At the same time, AI systems are rapidly becoming embedded in everyday work activities, enabling employees to draft documents, analyze information, generate content, write code, and refine outputs with unprecedented speed. These developments are widely viewed as productivity-enhancing innovations because they reduce repetitive effort and increase efficiency. The assumption that follows appears straightforward: if AI reduces workload, employees should find it easier to disengage from work and recover after working hours. Existing evidence suggests that the relationship is more complex. Recovery research has consistently shown that employees often struggle to achieve psychological detachment when work-related concerns remain cognitively active beyond formal working hours (Sonnentag and Fritz, 2007, 2015). Studies of hybrid work likewise indicate that flexibility frequently coexists with after-hours cognitive activation and work–life interference rather than automatically improving recovery (Pensar and Mäkelä, 2023; Wang et al., 2023). Emerging research on AI-enabled work points in a similar direction, suggesting that technological assistance can simultaneously enhance performance and introduce new forms of uncertainty, responsibility, and concern (Jarrahi, 2018; Parker and Grote, 2022). Despite these advances, existing explanations of recovery failure continue to focus primarily on workload, unfinished tasks, communication demands, and digital connectivity. Much less attention has been given to a different possibility emerging in AI-assisted work environments: employees may disengage from task execution while remaining psychologically attached to work processes that continue through AI-assisted systems. In such situations, the challenge is not simply that employees continue working after hours, but that delegated work remains cognitively open and psychologically active even after direct task execution has ended. Understanding this form of post-work cognitive attachment has become increasingly important as AI-assisted work becomes a routine feature of hybrid work arrangements. Studies of boundary management show that flexibility can increase role permeability when employees lack clear segmentation routines (Ashforth et al., 2000; Kossek et al., 2012). Remote and hybrid work research similarly links flexibility with recovery challenges when work remains cognitively active after hours (Wang et al., 2021; Pensar and Mäkelä, 2023). Work-family boundary research also suggests that positive flexibility and conflict can coexist within the same work arrangement (Chen et al., 2009; Allen et al., 2014). Connectivity studies further show that after-hours work behavior can undermine detachment and increase emotional exhaustion (Hu et al., 2024; Jin et al., 2026).

### 1.2 AI delegation, process vigilance, and the hidden cost of productivity

The growing use of AI in knowledge work is changing the nature of delegation. Traditional delegation typically involves transferring work to another person who assumes at least part of the responsibility for execution, monitoring, and adjustment. AI-assisted delegation differs in important ways because employees may transfer task execution to an intelligent system while remaining accountable for the quality, accuracy, and consequences of the resulting outputs. AI can draft reports, generate analyses, summarize information, write code, translate content, and produce recommendations without continuous human involvement, yet employees often remain responsible for verifying whether those outputs are correct, complete, and appropriate. Recent research on human–AI collaboration suggests that AI rarely functions as a complete substitute for human judgment; instead, it redistributes responsibility, oversight, and decision making across human and technological actors (Jarrahi, 2018; Faraj et al., 2018). As a result, employees may no longer be actively performing a task while continuing to think about how the delegated process is unfolding. Concerns about whether AI has misunderstood instructions, overlooked important context, generated inaccurate information, produced low-quality outputs, introduced hallucinated content, or created errors that will require later correction can remain psychologically salient long after direct task execution has ended. This pattern suggests a paradox at the center of AI-assisted work. The same technology that reduces workload and accelerates productivity may simultaneously create new monitoring demands by increasing uncertainty regarding process quality, progress, and outcomes. The present article refers to this tension as the AI delegation paradox. Employees may disengage from task performance while remaining cognitively attached to delegated work processes that continue beyond their direct supervision. To explain this phenomenon, the study introduces AI process vigilance (AIPV), defined as a post-delegation cognitive state characterized by sustained monitoring, concern, uncertainty, and attentional focus directed toward AI-assisted work processes and outputs outside formal working hours. AIPV is not concern about technology itself, nor is it simply a form of general technostress, digital overload, or work-related rumination. Rather, it reflects concern about delegated work that continues through technology while accountability remains with the employee. Individuals experiencing AIPV remain attentive to the status, quality, and potential consequences of AI-assisted work even when they are no longer directly engaged in task execution, making it possible for AI-enabled efficiency gains to coexist with persistent cognitive activation, difficulties achieving psychological detachment, and impaired recovery after work. Early organizational accounts of AI emphasized productivity and decision support, whereas later work highlights human-AI symbiosis in judgment and responsibility (Davenport and Ronanki, 2018; Jarrahi, 2018). Phenomenon-based AI research similarly argues that organizational AI changes how work is coordinated and theorized (Faraj et al., 2018; von Krogh, 2018). Research on AI decision structures shows that automation can redistribute authority while preserving human accountability (Shrestha et al., 2019; Glikson and Woolley, 2020). Algorithmic management studies further indicate that digital systems can create new forms of control and worker agency tension (Kellogg et al., 2020; Duggan et al., 2023). Recent HRM scholarship extends this point by showing that AI changes both strategic work design and human resource systems (Chowdhury et al., 2024; Kim et al., 2025).

### 1.3 Extending recovery theory through the AI delegation paradox

The emergence of AI process vigilance raises a broader theoretical question concerning how recovery should be understood in AI-assisted work environments. Recovery research has traditionally emphasized the importance of psychological detachment, arguing that employees recover when work-related cognitive activation subsides during nonwork time (Sonnentag and Fritz, 2007, 2015). Boundary theory similarly suggests that well-being depends on the ability to maintain meaningful separation between work and nonwork domains (Ashforth et al., 2000; Kossek et al., 2012). Conservation-of-resources theory proposes that continued cognitive demands can deplete valuable psychological resources even when visible work effort has ceased (Hobfoll, 1989). These perspectives have substantially advanced understanding of recovery, yet they were developed largely in contexts where work was assumed to remain psychologically active because employees continued performing tasks, responding to communications, or managing unfinished responsibilities. AI-assisted work introduces a different possibility. Employees may stop executing tasks while remaining cognitively engaged with delegated work processes that continue through intelligent systems. Under these conditions, psychological activation originates not from ongoing task performance but from continued monitoring, uncertainty, and accountability regarding delegated work. This argument extends existing recovery theory by suggesting that recovery impairment may arise from delegated-process monitoring rather than continued work activity alone. It also contributes to boundary research by highlighting how work can remain psychologically present even when employees are not actively interacting with work technologies, and it extends conservation-of-resources theory by identifying a form of vigilance-based resource drain that emerges when individuals remain concerned about work processes operating beyond their direct control. Building on these perspectives, the present study develops the AI delegation paradox and proposes a serial process in which continuous AI availability encourages greater AI delegation dependence, delegation dependence increases AI process vigilance, vigilance promotes work-related rumination, rumination undermines psychological detachment, and reduced detachment ultimately impairs recovery outcomes. The conceptual model therefore seeks to explain not only how AI-assisted work affects recovery, but also why technologies designed to reduce effort may simultaneously create new psychological barriers to disengagement and restoration. Meta-analytic recovery evidence shows that detachment and recovery experiences are central to well-being after work (Bennett et al., 2018; Wendsche and Lohmann-Haislah, 2017). Telepressure and mobile-email research shows that digital tools can keep employees psychologically attached to work even without formal work time (Barber and Santuzzi, 2015; Mazmanian et al., 2013). Work rumination research further explains why unresolved work concerns can persist beyond the work episode (Cropley and Zijlstra, 2011; Querstret and Cropley, 2012). Classic effort-recovery and resource theories suggest that strain can emerge when recovery resources are continuously threatened (Meijman and Mulder, 1998; Hobfoll, 1989).

### 1.4 Theoretical contributions

The emergence of AI-assisted work raises a broader theoretical question concerning how recovery should be understood when task execution and responsibility become partially separated. Existing constructs such as technostress, telepressure, work connectivity, and work-related rumination capture important forms of technology-related strain, response pressure, boundary permeability, and repetitive cognition, yet they do not fully explain the post-delegation state in which employees disengage from direct task execution while remaining psychologically attached to work processes that continue through AI systems. To address this gap, the present study introduces AI process vigilance (AIPV), a construct that captures sustained attention, monitoring, uncertainty, and concern directed toward delegated AI-assisted work outside formal working hours. By focusing on progress monitoring, output-quality concern, error anticipation, and perceived loss of control, AIPV explains why AI-enabled efficiency may remain psychologically active even after work has ostensibly ended. This perspective extends recovery theory by suggesting that recovery impairment may arise not only from continued work activity, after-hours communication, or unfinished tasks, but also from continued monitoring of delegated work processes. Employees may no longer be performing a task, yet remain concerned about whether an AI system is functioning appropriately, whether outputs will require correction, and whether accountability will ultimately return to them. The study further contributes to conservation-of-resources theory by identifying a form of vigilance-based resource drain in which execution effort declines while cognitive resources continue to be consumed by uncertainty, oversight, and responsibility for outcomes that are no longer fully under direct control. In this sense, AI-assisted work creates a paradox: technologies designed to reduce effort may simultaneously generate new psychological barriers to detachment and recovery. Building on these arguments, the article develops the AI delegation paradox and proposes a serial process in which continuous AI availability increases dependence on AI-assisted delegation, delegation dependence heightens AI process vigilance, vigilance promotes work-related rumination, rumination undermines psychological detachment, and reduced detachment ultimately impairs recovery outcomes. The resulting framework offers an integrated explanation for how AI-assisted work reshapes the relationship between productivity, cognitive activation, and recovery in contemporary hybrid work environments. Technostress theory provides one foundation for this argument, but it does not fully capture delegated-process monitoring (Tarafdar et al., 2007; Ayyagari et al., 2011). AI-specific technostress studies show that AI adoption can trigger anxiety, uncertainty, and ambivalent affective reactions (Chang et al., 2024; Zhang et al., 2025). Recent reviews also suggest that workplace AI can simultaneously improve efficiency and create new employee well-being risks (Soulami et al., 2024; Everson, 2025). Workplace AI studies further show that AI use is associated with employee affective well-being and broader well-being outcomes (Jin et al., 2024; Valtonen et al., 2025).

## 2 Theory and hypotheses

### 2.1 The AI delegation paradox

The growing adoption of AI in knowledge work is changing the nature of delegation. Traditional delegation typically involves transferring work to another person who assumes at least part of the responsibility for execution, monitoring, and adjustment, whereas AI-assisted delegation allows employees to transfer execution to an intelligent system while remaining accountable for the quality, appropriateness, and consequences of the resulting outputs. AI can draft reports, generate analyses, summarize information, write code, and produce recommendations without continuous human involvement, yet responsibility for evaluating and ultimately approving those outputs often remains with the employee. As a result, delegation may reduce direct effort without fully eliminating concern about the delegated work itself. This asymmetry creates the foundation for what the present study refers to as the AI delegation paradox. AI is frequently promoted as a tool that reduces workload and increases efficiency, but the same technology may simultaneously create new monitoring demands because employees remain responsible for work processes that continue beyond their direct supervision. They may no longer be actively performing a task while continuing to think about whether the delegated process is progressing appropriately, whether important information has been overlooked, whether outputs will satisfy organizational standards, and whether errors will later require correction. In this sense, AI-assisted work can reduce execution demands while increasing uncertainty regarding process quality, progress, and outcomes. The benefits of delegation therefore coexist with a continuing sense of responsibility for work that remains only partially visible to the employee. The likelihood that employees rely on AI-assisted delegation is further shaped by the extent to which AI remains continuously available as a work resource. When AI tools are accessible across tasks, locations, and work periods, employees are more likely to integrate them into routine work processes and transfer a greater proportion of task execution to AI-assisted systems. Continuous availability lowers the practical barriers to delegation, encourages reliance on AI-supported execution, and increases the extent to which work activities become embedded in AI-assisted workflows. Employees who can access AI whenever needed are therefore more likely to develop dependence on AI-assisted delegation as a regular feature of their work routines, creating conditions under which responsibility remains with the employee even as execution increasingly shifts to AI systems.

*H1*: Continuous AI availability is positively associated with AI delegation dependence.

AI delegation dependence is not inherently problematic. Dependence reflects the successful integration of AI into work processes and may improve efficiency, speed, and productivity. However, dependence becomes psychologically consequential when delegated work remains uncertain, open, and subject to later evaluation. AI-generated outputs often require verification, interpretation, and quality assessment before they can be fully trusted or implemented. Employees who rely more heavily on AI-assisted delegation may therefore become increasingly attentive to whether delegated work is progressing appropriately and whether the resulting outputs will ultimately meet expectations. This continuing attentional attachment to delegated work creates the conditions under which vigilance emerges. Greater dependence on AI-assisted delegation should therefore increase the likelihood that employees remain cognitively engaged with delegated work processes even after direct task execution has ended. Algorithmic management research shows that digital delegation and control can have mixed consequences for autonomy and well-being (Felix et al., 2023; Lu et al., 2024). Platform and algorithmic attribution studies similarly show that algorithmic systems can create both operational benefits and psychological costs (Zhou et al., 2025; Dasgupta et al., 2026).

*H2*: AI delegation dependence is positively associated with AI process vigilance.

### 2.2 AI process vigilance as a distinct post-delegation cognitive state

The AI delegation paradox suggests that employees may remain psychologically attached to work even after direct task execution has ended. Explaining this phenomenon requires a construct that captures the unique cognitive state created when delegated work continues through AI-assisted systems while accountability remains with the employee. The present study introduces AI process vigilance (AIPV) to describe this state. AIPV refers to a post-delegation condition characterized by sustained monitoring, concern, uncertainty, and attentional focus directed toward AI-assisted work processes and outputs outside formal working hours. Unlike traditional forms of technology-related strain, AIPV is not primarily concerned with technology itself. Employees experiencing AIPV are not necessarily worried about learning a system, adapting to technological complexity, responding to messages, or remaining digitally connected. Instead, their attention is directed toward the status, quality, and potential consequences of delegated work that continues through AI-assisted processes. This distinction separates AIPV from several adjacent constructs. Technostress reflects strain arising from technology characteristics such as complexity, overload, invasion, or uncertainty, whereas AIPV concerns delegated work outcomes rather than technology use itself. Telepressure captures the urge to respond quickly to work-related communications, whereas AIPV can emerge even in the absence of messages or interpersonal demands because the object of concern is the delegated work process. Availability stress reflects expectations of accessibility, while AIPV reflects concern about accountability for work that remains in progress. Work-related rumination captures repetitive work-related thought more broadly, but AIPV represents an upstream vigilance state that may increase the likelihood that delegated-process concerns become the subject of repetitive thinking. The distinction is important because the object of concern changes the underlying mechanism. If recovery difficulties originate from technology strain, interventions should focus on usability and complexity reduction. If they originate from communication pressure, interventions should target responsiveness norms and boundary management. If they originate from workload, reducing task demands may be sufficient. AIPV suggests a different source of recovery impairment. Employees may value AI assistance, appreciate the productivity benefits it provides, and still remain cognitively attached to delegated work because they are uncertain about whether the process is unfolding appropriately and whether responsibility will ultimately return to them. Questions about progress, output quality, hidden errors, hallucinated content, overlooked information, and loss of oversight can remain psychologically active even after direct task execution has stopped. This process-accountability concern represents the defining characteristic of AIPV and explains why AI-assisted work may remain cognitively open long after formal working hours have ended. Recent AI-work studies indicate that employees may engage with AI productively while still experiencing concern about skill, control, and accountability (Guo and Wei, 2025; Wang et al., 2026). Studies of generative AI use also suggest that job crafting, engagement, and responsible adoption depend on how employees integrate AI into work routines (Meng et al., 2023; Yi et al., 2025).

Table 1 summarizes the conceptual distinctions between AIPV and related technology, boundary, and recovery constructs.

**Table 1:** Distinguishing AIPV from related constructs.

Construct	Primary object	Temporal focus	Underlying mechanism	Why different from AIPV
Technostress	Technology demands	During and around use	Strain from technology complexity, overload, invasion, or uncertainty	AIPV concerns delegated work outcomes continuing through AI, not technology strain in general
Telepressure	Messages and response expectations	After-hours and between tasks	Urge to respond quickly to communication	AIPV is not response pressure; it is monitoring of AI-assisted work progress and output quality
Availability stress	Being reachable	Boundary-crossing periods	Stress from expected accessibility	AIPV may occur without others contacting the employee because the AI process itself remains open
Digital connectivity	Device and platform connection	Continuous	Connectivity enables work contact	AIPV focuses on delegated work uncertainty after AI has been used
Boundary permeability	Role boundaries	Work-nonwork transitions	Movement of work concerns across domains	AIPV is a specific cognitive content that can permeate boundaries
Workload	Task volume and demands	Work time	Effort expenditure and demand intensity	AIPV may increase even when direct task execution declines
AI anxiety	AI as threatening technology	General future or work context	Fear of AI replacement, competence loss, or disruption	AIPV concerns accountability for AI-assisted work outputs, not broad fear of AI
Algorithmic control	Algorithmic management systems	During managed work	Monitoring, evaluation, and control by algorithms	AIPV concerns employees monitoring AI-delegated work, not being controlled by algorithms
Work-related rumination	Work problems generally	Nonwork time	Repetitive work-related thinking	AIPV is an upstream, AI-specific vigilance state that can trigger rumination

### 2.3 AI process vigilance, rumination, and recovery

The central implication of AI process vigilance is that delegated work may remain cognitively active even when employees are no longer directly engaged in task execution. Recovery research has consistently emphasized the importance of psychological detachment, arguing that employees recover most effectively when work-related thoughts and concerns no longer occupy attention during nonwork time (Sonnentag and Fritz, 2007, 2015). AIPV introduces a potential obstacle to this process because delegated AI-assisted work remains psychologically open rather than cognitively completed. Employees who remain concerned about whether AI-generated outputs are accurate, whether delegated processes are progressing appropriately, and whether future corrections will be required may repeatedly revisit these concerns during nonwork hours. Such repeated cognitive engagement is conceptually consistent with work-related rumination, which refers to persistent and recurring work-related thoughts that continue after formal work has ended (Cropley and Zijlstra, 2011; Querstret and Cropley, 2012). AIPV therefore represents a likely antecedent of rumination because vigilance keeps delegated work psychologically salient and increases the probability that employees continue thinking about work-related issues outside working hours. Recent work on AI collaboration suggests that leader support and human-centered design can shape whether AI use becomes empowering or draining (Meng et al., 2025; Reinhard et al., 2023). Research on AI adoption and workplace training further highlights that integration practices matter for whether employees experience AI as a resource or demand (Muehlemann, 2024; Bharti, 2025).

*H3*: AI process vigilance is positively associated with work-related rumination.

The extent to which vigilance develops into rumination may depend on how employees manage the boundary between work and nonwork domains. Boundary theory suggests that individuals differ in their preferences for separating or integrating work and personal life (Ashforth et al., 2000; Kossek et al., 2012). Employees with stronger boundary management preferences are generally more motivated to protect nonwork time from work-related intrusion and may therefore be better able to limit the extent to which vigilance-related concerns become repetitive work-related thought. Although vigilance may arise because delegated work remains uncertain, individuals who prefer clearer work–nonwork separation may be more effective at preventing those concerns from dominating attention outside formal working hours.

*H4*: Boundary management preference weakens the positive association between AI process vigilance and work-related rumination.

Work related rumination is expected to interfere with psychological detachment because repeated attention to work-related concerns prevents employees from mentally disengaging from work demands. Detachment represents a core recovery experience through which employees temporarily suspend work-related cognitive activation and replenish depleted resources (Sonnentag and Fritz, 2007). Employees who continue revisiting work-related thoughts are less able to achieve this psychological separation, making effective recovery more difficult. Lower detachment should therefore represent a key mechanism through which delegated-process vigilance ultimately affects employee well-being.

*H5*: Work-related rumination is negatively associated with psychological detachment.

Recovery theory and conservation-of-resources theory both suggest that psychological detachment supports restoration by reducing continuing activation and protecting personal resources (Hobfoll, 1989; Sonnentag and Fritz, 2015). Employees who successfully detach from work are more likely to experience psychological recovery, whereas employees who remain cognitively engaged with work concerns may experience continued resource depletion. In AI-assisted work environments, this process implies that vigilance-related concerns can indirectly impair recovery by increasing rumination and reducing detachment.

*H6*: Psychological detachment is positively associated with recovery outcomes.

Taken together, the proposed model suggests a serial process linking AI-assisted work to recovery outcomes. Continuous AI availability encourages greater reliance on AI-assisted delegation, delegation dependence increases AI process vigilance, vigilance promotes work-related rumination, rumination undermines psychological detachment, and reduced detachment impairs recovery. The resulting sequence represents the core mechanism through which the AI delegation paradox may translate into after-hours recovery costs.

*H7*: Continuous AI availability has a serial indirect association with recovery outcomes through AI delegation dependence, AI process vigilance, work-related rumination, and psychological detachment.

## 3 Method

### 3.1 Participants and procedure

The study employed a two-wave survey design with a seven-week interval to reduce concerns regarding temporal proximity among the focal constructs. Participants were recruited from knowledge-intensive occupations in which AI-assisted work was routinely used. Eligibility required respondents to use AI tools for work-related purposes at least once per week and to work in a hybrid or predominantly remote arrangement. At Wave 1, participants reported continuous AI availability, AI delegation dependence, boundary management preference, and demographic information. Seven weeks later, the same participants completed measures of AI process vigilance, work-related rumination, psychological detachment, and recovery outcomes. A total of 742 respondents completed the first survey. After matching responses across waves and removing incomplete cases, the final sample consisted of 642 participants, representing a retention rate of 86.5%. Participants were employed across a range of professional and knowledge-intensive occupations, including business services, information technology, education, consulting, finance, and administrative work. The average age of participants was 35.52 years (SD = 7.54), organizational tenure averaged 6.47 years (SD = 3.26), and 50.8% of respondents were female. Table 2 presents the demographic characteristics of the sample.

**Table 2:** Sample characteristics and attrition.

Variable	Category	Value
T1 respondents	*N*	742
T2 retained	*N* (%)	642 (86.5%)
Time lag	Weeks	7
Age at T1	Mean (SD)	35.52 (7.54)
Organizational tenure	Mean (SD)	6.47 (3.26)
AI use days per week	Mean (SD)	3.93 (1.18)
Remote work days per week	Mean (SD)	2.91 (0.98)
Gender	Female	326
Gender	Male	304
Gender	Nonbinary/prefer not to say	12
Work arrangement	Hybrid	503
Work arrangement	Mostly remote	139
Industry	Administration/operations	120
Industry	Consulting	92
Industry	Education/research	86
Industry	Finance/accounting	112
Industry	IT/digital services	142
Industry	Marketing/design	90

### 3.2 Measures and analysis

All focal constructs were measured using multi-item scales assessed on five-point Likert-type response formats ranging from 1 (strongly disagree) to 5 (strongly agree), with higher scores indicating higher levels of the underlying construct. Continuous AI availability was measured using four items assessing the extent to which AI tools remained readily accessible across work tasks and work situations (*α* = 0.814). AI delegation dependence was measured using five items capturing the degree to which employees relied on AI systems to execute work-related tasks that would otherwise require direct personal effort (*α* = 0.850). Boundary management preference was assessed with four items measuring employees’ preferences for maintaining separation between work and nonwork domains (*α* = 0.776). AI process vigilance (AIPV), the central construct of the study, was measured using 12 items representing four theoretically derived dimensions: progress monitoring, output quality concern, error anticipation, and loss of control. Consistent with the conceptualization of AIPV as a higher-order construct, the four dimensions were modeled as indicators of a common vigilance factor, and the overall scale demonstrated strong reliability (*α* = 0.914). Work-related rumination was measured with five items assessing repetitive work-related thoughts during nonwork time (*α* = 0.839), whereas psychological detachment was measured using four items capturing employees’ ability to mentally disengage from work during nonwork hours (*α* = 0.796). Recovery outcomes were assessed with five items reflecting restoration, replenishment of psychological resources, and readiness for subsequent work demands (*α* = 0.841). To evaluate the robustness of the proposed model, sleep quality and emotional exhaustion were additionally measured as alternative recovery-related outcomes, yielding acceptable reliability estimates (*α* = 0.735 and *α* = 0.750, respectively). Reliability, convergent validity, and discriminant validity statistics for all measures are reported in subsequent analyses.

### 3.3 Measurement validation

A series of analyses was conducted to evaluate the reliability and validity of the measurement model. As shown in Table 3, all focal constructs demonstrated satisfactory internal consistency and convergent validity. Cronbach’s α values ranged from 0.735 to 0.914, composite reliability (CR) values ranged from 0.834 to 0.927, and all average variance extracted (AVE) values exceeded the recommended threshold of 0.50. AI process vigilance exhibited particularly strong reliability (α = 0.914, CR = 0.927) despite representing a multidimensional second-order construct. Detailed item-level loadings are reported in Appendix Table A1.

**Table 3:** Reliability and convergent validity.

Construct	Wave	Items	Cronbach’s α	CR	AVE
Continuous AI availability	T1	4	0.814	0.878	0.643
AI delegation dependence	T1	5	0.850	0.893	0.626
Boundary management preference	T1	4	0.776	0.856	0.598
AI process vigilance	T2	12	0.914	0.927	0.515
Work-related rumination	T2	5	0.839	0.886	0.609
Psychological detachment	T2	4	0.796	0.867	0.620
Recovery outcomes	T2	5	0.841	0.887	0.611
Sleep quality robustness outcome	T2	4	0.735	0.834	0.557
Emotional exhaustion robustness outcome	T2	4	0.750	0.842	0.571
Marker attention	T2	3	0.637	0.805	0.579

Discriminant validity was assessed using the heterotrait-monotrait ratio (HTMT). As shown in Table 4, all HTMT values remained below commonly recommended thresholds, with the highest value observed between AI delegation dependence and AI process vigilance (HTMT = 0.628). The remaining values were substantially lower, providing evidence that the focal constructs are empirically distinct despite their theoretical relatedness. These findings support the discriminant validity of the measurement model and suggest that the constructs capture separate aspects of AI-assisted work, vigilance, rumination, detachment, and recovery.

**Table 4:** HTMT evidence.

Construct	Continuous AI availability	AI delegation dependence	Boundary management preference	AI process vigilance	Work-related rumination	Psychological detachment	Recovery outcomes	Marker attention
Continuous AI availability	1.000	0.589	0.046	0.518	0.196	0.124	0.129	0.127
AI delegation dependence	0.589	1.000	0.045	0.628	0.294	0.150	0.235	0.094
Boundary management preference	0.046	0.045	1.000	0.044	0.169	0.216	0.205	0.060
AI process vigilance	0.518	0.628	0.044	1.000	0.514	0.225	0.398	0.089
Work-related rumination	0.196	0.294	0.169	0.514	1.000	0.486	0.599	0.067
Psychological detachment	0.124	0.150	0.216	0.225	0.486	1.000	0.660	0.113
Recovery outcomes	0.129	0.235	0.205	0.398	0.599	0.660	1.000	0.089
Marker attention	0.127	0.094	0.060	0.089	0.067	0.113	0.089	1.000

Table 5 provides additional evidence using the Fornell–Larcker criterion, where the square root of the AVE for each construct exceeded its correlations with all other constructs. The distinctiveness of AI process vigilance is particularly important because the construct is conceptually related to delegation dependence and work-related rumination, yet remained empirically separable from both. Measurement model comparisons were also conducted to evaluate alternative construct structures.

**Table 5:** Fornell-Larcker evidence.

Construct	Continuous AI availability	AI delegation dependence	Boundary management preference	AI process vigilance	Work-related rumination	Psychological detachment	Recovery outcomes
Continuous AI availability	0.802	0.490	−0.007	0.447	0.162	−0.100	−0.107
AI delegation dependence	0.490	0.791	−0.014	0.553	0.248	−0.123	−0.199
Boundary management preference	−0.007	−0.014	0.773	0.011	−0.137	0.171	0.166
AI process vigilance	0.447	0.553	0.011	0.718	0.450	−0.192	−0.349
Work-related rumination	0.162	0.248	−0.137	0.450	0.780	−0.398	−0.503
Psychological detachment	−0.100	−0.123	0.171	−0.192	−0.398	0.787	0.540
Recovery outcomes	−0.107	−0.199	0.166	−0.349	−0.503	0.540	0.782

As reported in Table 6, the hypothesized two-wave seven-factor measurement model demonstrated excellent fit to the data (χ^2^ = 683.76, df = 681, χ^2^/df = 1.00, CFI = 1.000, TLI = 1.000, RMSEA = 0.003, SRMR = 0.028), substantially outperforming both the merged AI delegation–AIPV model (CFI = 0.868, RMSEA = 0.055) and the single-factor model (CFI = 0.555, RMSEA = 0.100). These comparisons provide strong evidence for the distinctiveness of the focal constructs and reduce concerns regarding common method bias. In addition, the second-order AIPV dimension model demonstrated excellent fit (χ^2^ = 3.26, df = 2, CFI = 0.999, TLI = 0.998, RMSEA = 0.031, SRMR = 0.008), supporting the conceptualization of AI process vigilance as a multidimensional yet coherent higher-order construct composed of progress monitoring, output quality concern, error anticipation, and loss of control. Taken together, the reliability, convergent validity, discriminant validity, and model-comparison results indicate that the measurement model provides a robust representation of the underlying constructs and offers a sound basis for subsequent hypothesis testing.

**Table 6:** Measurement model comparisons.

Model	χ^2^	df	χ^2^/df	CFI	TLI	RMSEA	SRMR
Two-wave seven-factor model	683.76	681	1.00	1.000	1.000	0.003	0.028
Second-order AIPV dimension model	3.26	2	1.63	0.999	0.998	0.031	0.008
Merged AI delegation and AIPV	2005.33	687	2.92	0.868	0.858	0.055	0.067
Single-factor model	5165.16	702	7.36	0.555	0.530	0.100	0.118

### 3.4 Second-order structure of AI process vigilance

AI process vigilance was conceptualized as a higher-order construct comprising four related dimensions: progress monitoring, output quality concern, error anticipation, and loss of control. This specification reflects the theoretical assumption that employees may experience different manifestations of vigilance while remaining psychologically attached to delegated AI-assisted work. A second-order confirmatory factor model was therefore estimated to examine whether the four dimensions reflected a common higher-order vigilance construct. As shown in Figure 1 and Table 7, all four first-order dimensions demonstrated acceptable internal consistency and loaded strongly on the higher-order AIPV factor. Standardized loadings ranged from 0.883 to 0.891. The second-order model demonstrated excellent fit to the data (χ^2^ = 3.26, df = 2, χ^2^/df = 1.63, CFI = 0.999, TLI = 0.998, RMSEA = 0.031, SRMR = 0.008), providing strong support for the conceptualization of AIPV as a multidimensional yet coherent higher-order construct. These findings justify the use of an overall AIPV score in subsequent analyses while preserving the theoretical interpretation that vigilance may be expressed through multiple forms of post-delegation concern.

**Figure 1:**
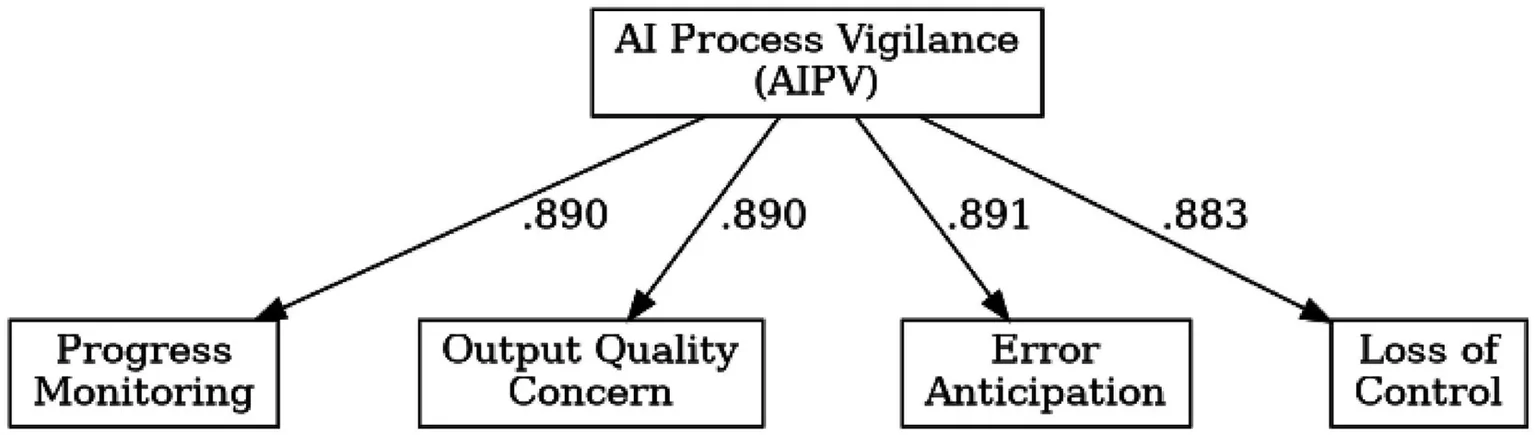
Second-order factor structure of AI process vigilance. Note. Standardized second-order factor loadings are displayed. Progress Monitoring (*λ* = 0.890), Output Quality Concern (*λ* = 0.890), Error Anticipation (*λ* = 0.891), and Loss of Control (*λ* = 0.883) loaded strongly on the higher-order AI Process Vigilance (AIPV) construct. The second-order model demonstrated excellent fit to the data (*χ*^2^ = 3.26, *df* = 2, *χ*^2^ / *df* = 1.63, *CFI* = 0.999, *TLI* = 0.998, *RMSEA* = 0.031, *SRMR* = 0.008).

**Table 7:** Second-order AIPV evidence.

First-order dimension	Number of items	Cronbach’s α	Loading on AIPV	Interpretation
Progress monitoring	3	0.736	0.890	Retained
Output quality concern	3	0.735	0.890	Retained
Error anticipation	3	0.732	0.891	Retained
Loss of control	3	0.727	0.883	Retained
Second-order AIPV model	Four first-order dimensions	—	χ^2^ = 3.26; CFI = 0.999; TLI = 0.998; RMSEA = 0.031; SRMR = 0.008	Dynamically fitted

### 3.5 Analytical strategy

Data were analyzed using Python 3.12.13 with the NumPy (2.4.6), Pandas (3.0.3), SciPy (1.18.0), and OpenPyXL (3.1.5) libraries. NumPy and Pandas were used for data management, scale construction, matrix operations, and statistical preprocessing. Confirmatory factor analyses and model-fit statistics were estimated using maximum-likelihood optimization procedures implemented in SciPy. OpenPyXL was used to generate and export the empirical result tables directly from the item-level dataset. All descriptive statistics, reliability estimates, validity assessments, regression analyses, bootstrap confidence intervals, alternative-model comparisons, and robustness checks were dynamically reproduced from the raw survey data through a fully scripted analytical workflow, thereby enhancing computational transparency and reproducibility.

Hypotheses were tested using a path-analytic framework combining confirmatory factor analysis, ordinary least squares (OLS) regression, moderation analysis, and bootstrap-based mediation testing. The proposed model specified continuous AI availability as the exogenous variable, AI delegation dependence and AI process vigilance as sequential mediators, work-related rumination and psychological detachment as downstream psychological mechanisms, and recovery outcomes as the final outcome variable. Direct effects were estimated using OLS regression with HC3 heteroskedasticity-consistent robust standard errors. Indirect effects were evaluated using bias-corrected bootstrap confidence intervals based on 5,000 resamples. The hypothesized moderating role of boundary management preference was examined through an interaction term between AI process vigilance and boundary management preference. Several control variables, including age, gender, organizational tenure, AI-use frequency, remote-work frequency, and workload, were included in all regression equations. To evaluate the robustness of the findings, additional analyses were conducted using sleep quality and emotional exhaustion as alternative outcome variables. Alternative theoretical models were also estimated and compared with the hypothesized model to assess whether the proposed delegation–vigilance–rumination pathway provided a superior explanation of the data. Statistical significance was evaluated using two-tailed tests, and 95% confidence intervals are reported throughout the results section.

## 4 Results

As shown in Table 2, the final matched sample consisted of 642 employees who completed both survey waves, representing an 86.5% retention rate over the seven-week interval. Participants were regular users of AI-assisted work tools, reporting AI use on nearly 4 days per week on average. The sample was drawn primarily from hybrid work arrangements, with representation across information technology, finance, consulting, education, administration, and related knowledge-intensive occupations. These characteristics are consistent with the study’s focus on AI-assisted hybrid work and provide an appropriate context for examining AI delegation, post-work cognition, and recovery processes.

Table 8 presents the descriptive statistics for the focal study variables. Continuous AI availability (M = 3.46, SD = 0.87) and AI delegation dependence (M = 3.40, SD = 0.87) exhibited moderate mean levels at Wave 1, suggesting that AI-assisted work was a routine feature of participants’ work environments. At Wave 2, AI process vigilance also demonstrated a moderate mean level (M = 3.33, SD = 0.78), indicating that concerns regarding delegated AI-assisted work were neither rare nor extreme among respondents. Work-related rumination, psychological detachment, and recovery outcomes likewise displayed substantial variability across participants, with observed values spanning most of the response scale. Overall, the distributions suggest adequate variance for subsequent correlational, measurement, and structural analyses.

**Table 8:** Descriptive statistics.

Variable	Wave	M	SD	Min	Max
Continuous AI availability	T1	3.46	0.87	1.00	5.00
AI delegation dependence	T1	3.40	0.87	1.20	5.00
Boundary management preference	T1	3.37	0.87	1.00	5.00
AI process vigilance	T2	3.33	0.78	1.25	5.00
Work-related rumination	T2	3.19	0.87	1.00	5.00
Psychological detachment	T2	3.10	0.88	1.00	5.00
Recovery outcomes	T2	3.10	0.88	1.00	5.00

Table 9 presents the zero-order correlations among the focal study variables. The correlation pattern is broadly consistent with the proposed theoretical framework. Continuous AI availability was positively associated with AI delegation dependence (*r* = 0.490) and AI process vigilance (*r* = 0.447), suggesting that greater access to AI tools is accompanied by stronger reliance on AI-assisted task execution and heightened post-delegation monitoring. AI delegation dependence showed its strongest association with AI process vigilance (*r* = 0.553), providing preliminary support for the argument that reliance on AI-assisted work is linked to increased attentional attachment to delegated processes. AI process vigilance was positively related to work-related rumination (*r* = 0.450) and negatively related to both psychological detachment (*r* = −0.192) and recovery outcomes (*r* = −0.349). Work-related rumination demonstrated substantial negative associations with psychological detachment (*r* = −0.398) and recovery outcomes (*r* = −0.503), whereas psychological detachment was positively associated with recovery outcomes (*r* = 0.540). Overall, the observed correlations are consistent with the hypothesized sequence linking AI availability, delegation dependence, vigilance, rumination, detachment, and recovery. At the same time, the correlations remain well below conventional thresholds associated with severe construct overlap, providing preliminary support for the distinctiveness of the focal constructs.

**Table 9:** Correlations analysis.

Variable	Continuous AI availability	AI delegation dependence	Boundary management preference	AI process vigilance	Work-related rumination	Psychological detachment	Recovery outcomes
Continuous AI availability	1.000	0.490	−0.007	0.447	0.162	−0.100	−0.107
AI delegation dependence	0.490	1.000	−0.014	0.553	0.248	−0.123	−0.199
Boundary management preference	−0.007	−0.014	1.000	0.011	−0.137	0.171	0.166
AI process vigilance	0.447	0.553	0.011	1.000	0.450	−0.192	−0.349
Work-related rumination	0.162	0.248	−0.137	0.450	1.000	−0.398	−0.503
Psychological detachment	−0.100	−0.123	0.171	−0.192	−0.398	1.000	0.540
Recovery outcomes	−0.107	−0.199	0.166	−0.349	−0.503	0.540	1.000

Table 10 presents the results of the common method and collinearity diagnostics. Several complementary procedures were conducted to evaluate the potential influence of common method variance. The Harman single-factor diagnostic indicated that the first unrotated factor accounted for 22.1% of total item variance, substantially below the conventional 50% threshold. The marker-variable analysis revealed only weak associations between the marker construct and the focal study variables, with the highest correlation reaching 0.088. Full collinearity assessment further indicated that all variance inflation factors remained well below the recommended threshold of 3.50, with the largest VIF equal to 1.85. In addition, comparison between the hypothesized seven-factor measurement model and the single-factor model revealed substantial deterioration in model fit for the latter (ΔCFI = 0.445; ΔRMSEA = 0.097), providing further evidence that the observed covariance structure cannot be adequately explained by a single underlying factor. Taken together, these results do not support the presence of a dominant common method factor. Although common-source measurement can never be completely excluded, the combination of temporal separation between survey waves and multiple statistical diagnostics suggests that common method bias is unlikely to account for the observed pattern of relationships.

**Table 10:** Common method and collinearity checks.

Test	Result	Decision
Harman single-factor diagnostic	First factor = 22.1% of item variance	Dynamically computed from item correlation matrix
Marker-variable correlation	Highest marker-to-core construct *r* = 0.088	Dynamically computed marker check
Full collinearity VIF	Largest VIF = 1.85	Dynamically computed from structural predictors
Common latent factor comparison	ΔCFI = 0.445; ΔRMSEA = 0.097	Computed from fitted measurement models

Table 11 presents the structural path estimates for the hypothesized model. Continuous AI availability was positively associated with AI delegation dependence (*β* = 0.486, *p* < 0.001) and AI process vigilance (*β* = 0.233, *p* < 0.001). AI delegation dependence was positively associated with AI process vigilance (*β* = 0.426, *p* < 0.001). AI process vigilance was positively associated with work-related rumination (*β* = 0.411, *p* < 0.001), whereas boundary management preference was negatively associated with work-related rumination (*β* = −0.144, *p* < 0.001). The interaction between AI process vigilance and boundary management preference was not significant (*β* = −0.038, *p* = 0.242). Work-related rumination was negatively associated with psychological detachment (*β* = −0.392, p < 0.001), while the direct path from AI process vigilance to psychological detachment was not significant (*β* = −0.015, *p* = 0.708). Recovery outcomes were positively associated with psychological detachment (*β* = 0.402, *p* < 0.001) and negatively associated with both work-related rumination (*β* = −0.276, *p* < 0.001) and AI process vigilance (*β* = −0.147, *p* < 0.001). Overall, the results supported H1, H2, H3, H5, and H6, whereas H4 was not supported.

**Table 11:** Path estimates.

Outcome	Predictor	*β*	SE	*p*	Sig.	95% CI
AI delegation dependence	Continuous AI availability	0.486	0.032	<0.001	***	[0.424, 0.548]
AI process vigilance	AI delegation dependence	0.426	0.036	<0.001	***	[0.356, 0.497]
AI process vigilance	Continuous AI availability	0.233	0.037	<0.001	***	[0.161, 0.305]
Work-related rumination	AI process vigilance	0.411	0.035	<0.001	***	[0.342, 0.480]
Work-related rumination	Boundary management preference	−0.144	0.035	<0.001	***	[−0.213, −0.075]
Work-related rumination	AIPV × boundary preference	−0.038	0.032	*p* = 0.242		[−0.101, 0.025]
Psychological detachment	Work-related rumination	−0.392	0.039	<0.001	***	[−0.469, −0.316]
Psychological detachment	AI process vigilance	−0.015	0.041	p = 0.708		[−0.096, 0.066]
Recovery outcomes	Psychological detachment	0.402	0.032	<0.001	***	[0.339, 0.464]
Recovery outcomes	Work-related rumination	−0.276	0.040	<0.001	***	[−0.353, −0.198]
Recovery outcomes	AI process vigilance	−0.147	0.036	<0.001	***	[−0.218, −0.077]

Table 12 presents the bootstrap estimates for the indirect and conditional effects. Continuous AI availability exhibited a significant indirect association with AI process vigilance through AI delegation dependence (*β* = 0.207, 95% CI [0.165, 0.252]). The indirect pathway extending from continuous AI availability through AI delegation dependence and AI process vigilance to work-related rumination was also significant (*β* = 0.085, 95% CI [0.064, 0.109]). The indirect effect linking continuous AI availability to psychological detachment through AI delegation dependence, AI process vigilance, and work-related rumination was negative and significant (*β* = −0.033, 95% CI [−0.045, −0.024]). In addition, the complete serial indirect pathway from continuous AI availability to recovery outcomes through AI delegation dependence, AI process vigilance, work-related rumination, and psychological detachment was significant (*β* = −0.013, 95% CI [−0.019, −0.009]). Conditional effects indicated that the association between AI process vigilance and work-related rumination remained positive at low (*β* = 0.449, 95% CI [0.359, 0.535]), mean (*β* = 0.411, 95% CI [0.344, 0.478]), and high (*β* = 0.374, 95% CI [0.277, 0.468]) levels of boundary management preference. However, the index of moderated mediation was not significant (*β* = −0.038, 95% CI [−0.101, 0.022]). Overall, the serial indirect effects were supported, whereas the moderation hypothesis was not supported.

**Table 12:** Indirect effects and moderation.

Effect	Estimate	Boot SE	95% CI Lower	95% CI Upper	Result
CAA → AID→AIPV	0.207	0.022	0.165	0.252	Supported
CAA → AID→AIPV→WRR	0.085	0.012	0.064	0.109	Supported
CAA → AID→AIPV→WRR → PD	−0.033	0.006	−0.045	−0.024	Supported
CAA → AID→AIPV→WRR → PD → REC	−0.013	0.003	−0.019	−0.009	Supported
AIPV→WRR at Low boundary	0.449	0.045	0.359	0.535	Supported
AIPV→WRR at Mean boundary	0.411	0.035	0.344	0.478	Supported
AIPV→WRR at High boundary	0.374	0.049	0.277	0.468	Supported
Index of moderated path	−0.038	0.032	−0.101	0.022	Not supported

Table 13 presents that several robustness analyses were conducted to evaluate whether the observed relationships generalized across alternative outcome specifications. When sleep quality was used as the outcome, psychological detachment remained positively associated with favorable outcomes (*β* = 0.278, *p* < 0.001), whereas work-related rumination remained negatively associated with sleep quality (*β* = −0.200, *p* < 0.001). Notably, the direct association between AI process vigilance and sleep quality was not significant (*β* = −0.027, *p* = 0.492), suggesting that vigilance may influence sleep primarily through intermediate cognitive mechanisms rather than through a direct pathway. A similar pattern emerged for emotional exhaustion. Psychological detachment was associated with lower emotional exhaustion (*β* = −0.212, *p* < 0.001), whereas work-related rumination was associated with higher emotional exhaustion (*β* = 0.322, *p* < 0.001). AI process vigilance also demonstrated a positive association with emotional exhaustion (*β* = 0.180, *p* < 0.001), indicating that vigilance-related cognitive activation may contribute to strain-related outcomes. Additional analyses excluding all control variables produced results that were substantively unchanged from the primary model. Psychological detachment remained positively associated with recovery outcomes (*β* = 0.402, *p* < 0.001), whereas work-related rumination (*β* = −0.277, *p* < 0.001) and AI process vigilance (*β* = −0.147, *p* < 0.001) remained negatively associated with recovery. Taken together, these findings indicate that the proposed relationships are robust across alternative outcome variables and model specifications, increasing confidence that the reported effects do not depend on a single operationalization of recovery.

**Table 13:** Robustness checks.

Outcome	Predictor	β	SE	*p*	Sig.	95% CI
Sleep quality	Psychological detachment	0.278	0.038	<0.001	***	[0.204, 0.352]
Sleep quality	Work-related rumination	−0.200	0.043	<0.001	***	[−0.284, −0.116]
Sleep quality	AI process vigilance	−0.027	0.040	*p* = 0.492		[−0.106, 0.051]
Emotional exhaustion	Psychological detachment	−0.212	0.038	<0.001	***	[−0.286, −0.138]
Emotional exhaustion	Work-related rumination	0.322	0.042	<0.001	***	[0.239, 0.404]
Emotional exhaustion	AI process vigilance	0.180	0.039	<0.001	***	[0.104, 0.256]
Recovery without controls	Psychological detachment	0.402	0.032	<0.001	***	[0.340, 0.464]
Recovery without controls	Work-related rumination	−0.277	0.038	<0.001	***	[−0.352, −0.202]
Recovery without controls	AI process vigilance	−0.147	0.036	<0.001	***	[−0.217, −0.077]

Table 14 presents the alternative model comparisons. The hypothesized two-wave delegation–vigilance model demonstrated the strongest overall fit to the data (χ^2^ = 21.11, df = 10, χ^2^/df = 2.11, CFI = 0.989, TLI = 0.978, RMSEA = 0.042, SRMR = 0.037). In contrast, the direct AI availability model showed substantially poorer fit (CFI = 0.837, RMSEA = 0.156, SRMR = 0.139), indicating that AI availability alone did not adequately explain the observed relationships. Model fit deteriorated further when work-related rumination was removed from the theoretical sequence (CFI = 0.732, RMSEA = 0.192, SRMR = 0.170), suggesting that rumination represents an important psychological mechanism linking AI process vigilance to recovery-related outcomes. The reverse recovery model also demonstrated inferior fit (CFI = 0.862, RMSEA = 0.144, SRMR = 0.105). Taken together, the model-comparison results support the proposed delegation–vigilance–rumination pathway and indicate that the hypothesized model provides the most adequate representation of the observed covariance structure.

**Table 14:** Alternative model comparisons.

Model	Description	χ^2^	df	CFI	TLI	RMSEA	SRMR	Decision
Hypothesized two-wave delegation-vigilance model	T1 CAA/AID/BMP → T2 AIPV/WRR/PD/REC	21.11	10	2.11	0.989	0.978	0.042	0.037
Direct AI availability model	CAA predicts rumination and recovery without delegation/vigilance chain	183.64	11	16.69	0.837	0.688	0.156	0.139
No-rumination model	AIPV predicts detachment directly, omitting rumination	295.20	12	24.60	0.732	0.531	0.192	0.170
Reverse recovery model	Low recovery predicts rumination and AIPV	156.97	11	14.27	0.862	0.736	0.144	0.105

## 5 Discussion

### 5.1 AI delegation paradox and recovery theory

The principal theoretical contribution of the present study is the identification of a recovery mechanism that operates through continued monitoring rather than continued work. Existing recovery research has consistently emphasized that employees struggle to recover when they remain behaviorally or cognitively engaged with work after formal working hours, whether through unfinished tasks, excessive workload, persistent role demands, or ongoing work-related thought (Sonnentag and Fritz, 2007, 2015). The present findings suggest that AI-assisted hybrid work introduces a different pathway through which recovery may be undermined. Employees may successfully disengage from task execution while remaining psychologically attached to work that has been delegated to AI systems. The empirical results support this interpretation. Continuous AI availability was positively associated with AI delegation dependence, delegation dependence was positively associated with AI process vigilance, and AI process vigilance was strongly associated with work-related rumination (*β* = 0.411, *p* < 0.001). The complete indirect pathway linking AI availability to recovery outcomes through delegation dependence, vigilance, rumination, and psychological detachment was also significant (*β* = −0.013, 95% CI [−0.019, −0.009]). These findings indicate that recovery difficulties can emerge even when employees are no longer actively working because responsibility, uncertainty, and concern remain attached to delegated AI-assisted processes. This pattern reflects what the present study conceptualizes as the AI delegation paradox. AI is widely promoted as a productivity-enhancing technology because it reduces execution effort, accelerates task completion, and allows employees to offload routine cognitive work. However, delegation transfers execution without fully transferring accountability. Employees remain responsible for evaluating outputs, correcting mistakes, and managing the consequences of AI-generated work. As a result, they may continue thinking about whether AI has misunderstood instructions, overlooked relevant information, generated inaccurate content, introduced hallucinations, stalled during execution, or produced outputs that will later require revision. The psychological challenge is therefore not simply whether work continues, but whether employees can achieve cognitive closure regarding work that continues through AI after direct human involvement has ended.

The findings further extend recovery theory and conservation of resources theory by identifying vigilance-based resource drain as a distinct mechanism through which resources may be depleted. Resource loss is typically associated with effort expenditure, workload, role demands, or threats to valued resources (Hobfoll, 1989), yet the present results suggest that cognitive resources may also be consumed through monitoring, uncertainty, anticipatory concern, and accountability for delegated processes that remain psychologically open. Employees continue investing attention in work they are no longer performing directly because responsibility has not been fully relinquished. This observation broadens existing understandings of recovery and resource depletion by demonstrating that psychological activation can persist in the absence of visible task activity. In AI-assisted hybrid work, employees may leave work physically and behaviorally while remaining mentally connected to delegated processes whose quality, progress, and consequences remain uncertain. Recovery theory has traditionally focused on employees who cannot stop working; the present findings suggest that an equally important challenge may be employees who stop working but cannot stop supervising. This distinction becomes increasingly important as AI systems assume greater responsibility for knowledge-intensive tasks because the psychological burden of work may shift from execution to oversight. Organizations may therefore overestimate the recovery benefits of AI if they evaluate only time savings and task completion while overlooking the cognitive demands associated with delegated-process monitoring.

### 5.2 Construct contribution and boundary conditions

A second contribution of the study lies in the identification of AI process vigilance as a distinct mechanism linking AI-assisted work to recovery outcomes. Existing research has provided valuable explanations for technology-related strain, including technostress, telepressure, digital connectivity, availability pressure, and work-related rumination. These perspectives explain how technology complexity, communication expectations, constant accessibility, and repetitive work-related thought can interfere with employee well-being. However, they do not fully capture the psychological condition observed in the present study. AI process vigilance is not primarily concern about technology itself, pressure to respond to others, or repetitive reflection on completed work. Rather, it reflects sustained attention toward a delegated work process that continues through AI while responsibility remains with the employee. The object of concern is therefore different. Employees experiencing AI process vigilance are not necessarily worried about using technology, receiving messages, or handling excessive workload. Instead, they remain attentive to whether AI-assisted work is progressing correctly, whether outputs will meet quality expectations, whether hidden errors may emerge, and whether future correction will be required. This distinction becomes increasingly important as AI systems assume a larger role in knowledge work because AI differs from traditional communication technologies in its ability to continue producing outputs after direct human execution has ended. The employee may no longer be working, yet the delegated process remains psychologically active because accountability has not disappeared. The empirical evidence supports this conceptual distinction. AI process vigilance remained empirically separable from delegation dependence and work-related rumination in the validity analyses, and the hypothesized measurement model demonstrated substantially better fit than alternative merged-factor specifications. The findings also clarify how AI process vigilance operates. Boundary management preference was negatively associated with work-related rumination, indicating that employees who prefer stronger separation between work and nonwork generally experience fewer intrusive work-related thoughts. However, the hypothesized moderation effect was not supported.

Although the conditional estimates followed the expected direction, the confidence interval for the moderated effect included zero, suggesting insufficient evidence that stable boundary preferences substantially alter the pathway from vigilance to rumination. One possible explanation is that vigilance is driven less by dispositional preferences and more by situational features of delegated work, including task importance, output uncertainty, accountability expectations, and perceived consequences of AI failure. The non-significant direct effect from AI process vigilance to psychological detachment provides further insight into the underlying mechanism. Vigilance alone did not directly reduce detachment once rumination was included in the model, suggesting that concern about delegated AI-assisted work becomes recovery-relevant only when it develops into repetitive work-related thought. In other words, employees may monitor delegated processes without necessarily experiencing recovery difficulties, but recovery becomes impaired when vigilance evolves into persistent cognitive preoccupation. This interpretation is reinforced by the alternative model comparisons, which showed that models omitting rumination fit the data substantially worse than the hypothesized framework. Taken together, these findings suggest that AI process vigilance represents a distinct form of post-delegation cognitive attachment and that its consequences for recovery operate primarily through the generation of work-related rumination rather than through a direct effect on psychological detachment.

### 5.3 Practical implications

An additional contribution of the present study concerns the non-significant moderating effect of boundary management preference. Although boundary management preference was negatively associated with work-related rumination, indicating that employees who preferred stronger work–nonwork separation generally experienced lower levels of repetitive work-related thought, the interaction between boundary management preference and AI process vigilance was not significant. This finding suggests that stable segmentation preferences may not substantially weaken the relationship between vigilance and rumination in AI-assisted work. Existing boundary management research has generally assumed that employees can reduce after-hours cognitive activation by establishing clearer boundaries between work and nonwork domains. Such mechanisms are often effective when work-related cognition is driven by communication demands, availability expectations, unfinished tasks, or ongoing work activities. The present findings suggest that AI-assisted work introduces a different form of boundary challenge.

AI process vigilance originates from concern about delegated work processes that continue through AI while responsibility remains with the employee. Consequently, employees may continue thinking about delegated work even when they successfully separate themselves from work activities, communication channels, or physical workspaces. In this context, the central issue is not whether employees remain connected to work, but whether they feel psychologically released from accountability for delegated outputs. Boundary preferences may help employees disengage from work activities, yet they may be less effective in reducing concerns regarding work whose quality, progress, and consequences remain uncertain. This interpretation is consistent with the relatively strong association between AI process vigilance and work-related rumination (*β* = 0.411), compared with the non-significant interaction effect involving boundary management preference (*β* = −0.038, *p* = 0.242). The findings therefore suggest that accountability-related cognitive attachment may represent a stronger driver of rumination than segmentation preference in AI-assisted work contexts, reinforcing the broader argument that recovery difficulties can arise even after employees have stopped working because they remain psychologically attached to delegated AI-assisted processes.

### 5.4 Limitations and future research

Several limitations should be acknowledged. Although the study employed a two-wave design with a seven-week interval, the data remain observational and do not permit strong causal inference. Future research should use longitudinal, diary, and experimental designs to examine how AI process vigilance develops and changes over time. In addition, AI process vigilance is introduced as a new construct. While the present study provides evidence of reliability, validity, and a second-order factor structure, further validation using behavioral indicators, AI-use logs, digital trace data, and supervisor assessments would strengthen confidence in the construct. The sample was drawn primarily from hybrid and remote knowledge workers who regularly used AI-assisted tools, which may limit the generalizability of the findings to other occupations and organizational settings. Future studies should examine whether the proposed delegation, vigilance, rumination, and recovery mechanism operates similarly across industries, cultures, and levels of AI adoption. Despite these limitations, the study contributes a new perspective on employee recovery by demonstrating that the psychological consequences of AI extend beyond task automation and productivity enhancement. As AI becomes increasingly integrated into knowledge work, understanding how employees remain cognitively attached to delegated processes may become an important direction for future research.

## Data Availability

The raw data supporting the conclusions of this article will be made available by the authors, without undue reservation.

## Appendix

**Table A1:** Item-level loadings.

Construct	Wave	Item	Item wording	Standardized loading
Continuous AI availability	T1	Item 1	I can access AI tools whenever I need help with work tasks.	0.804
Continuous AI availability	T1	Item 2	AI tools are available even outside my formal working hours.	0.784
Continuous AI availability	T1	Item 3	AI makes it possible for work tasks to continue after hours.	0.811
Continuous AI availability	T1	Item 4	When a work-related issue arises after work, AI can immediately provide assistance.	0.807
AI delegation dependence	T1	Item 1	I rely on AI to continue or complete work-related tasks.	0.796
AI delegation dependence	T1	Item 2	I often delegate parts of my work to AI tools.	0.791
AI delegation dependence	T1	Item 3	AI has become an important support for completing my work.	0.780
AI delegation dependence	T1	Item 4	I depend on AI when refining work outputs.	0.802
AI delegation dependence	T1	Item 5	Some work tasks are difficult to complete efficiently without AI assistance.	0.786
Boundary management preference	T1	Item 1	I prefer to keep work and personal life separate.	0.763
Boundary management preference	T1	Item 2	I avoid work-related activities during personal time.	0.791
Boundary management preference	T1	Item 3	I prefer clear boundaries between working and nonworking hours.	0.769
Boundary management preference	T1	Item 4	I try not to let work interfere with my private time.	0.770
AI process vigilance	T2	Item 1	After work, I still think about the progress of AI-assisted tasks.	0.711
AI process vigilance	T2	Item 2	I feel a need to check whether AI has completed the task properly.	0.723
AI process vigilance	T2	Item 3	I often wonder how far AI has progressed on a work-related task.	0.729
AI process vigilance	T2	Item 4	I worry about the quality of AI-generated work outputs.	0.717
AI process vigilance	T2	Item 5	I think about whether AI outputs may contain errors after work.	0.714
AI process vigilance	T2	Item 6	I feel concerned that AI-generated content may not meet work standards.	0.729
AI process vigilance	T2	Item 7	I worry that AI may make mistakes without my awareness.	0.711
AI process vigilance	T2	Item 8	I am concerned that AI may misunderstand task requirements.	0.710
AI process vigilance	T2	Item 9	I think about possible AI errors even after I stop working.	0.736
AI process vigilance	T2	Item 10	I feel uneasy when AI continues a work process beyond my direct control.	0.713
AI process vigilance	T2	Item 11	I worry that AI may produce unexpected results.	0.694
AI process vigilance	T2	Item 12	I find it difficult to fully relax when work has been delegated to AI.	0.721
Work-related rumination	T2	Item 1	After work, I keep thinking about work-related tasks.	0.805
Work-related rumination	T2	Item 2	I find it difficult to stop thinking about work problems.	0.770
Work-related rumination	T2	Item 3	I repeatedly think about whether work tasks have been completed properly.	0.768
Work-related rumination	T2	Item 4	I keep thinking about unfinished work during nonwork time.	0.761
Work-related rumination	T2	Item 5	I find myself mentally returning to work even after the workday ends.	0.796
Psychological detachment	T2	Item 1	During nonwork time, I forget about work.	0.776
Psychological detachment	T2	Item 2	After work, I distance myself from work-related thoughts.	0.796
Psychological detachment	T2	Item 3	I do not think about work during my free time.	0.792
Psychological detachment	T2	Item 4	I mentally disconnect from work after working hours.	0.786
Recovery outcomes	T2	Item 1	I feel mentally recovered after nonwork time.	0.815
Recovery outcomes	T2	Item 2	I feel refreshed after resting.	0.748
Recovery outcomes	T2	Item 3	I wake up feeling restored.	0.788
Recovery outcomes	T2	Item 4	I feel energetic the next workday.	0.784
Recovery outcomes	T2	Item 5	I feel emotionally recovered from work demands.	0.774
Sleep quality robustness outcome	T2	Item 1	I slept well during the previous week.	0.744
Sleep quality robustness outcome	T2	Item 2	My sleep helped me recover from work demands.	0.758
Sleep quality robustness outcome	T2	Item 3	I woke up feeling rested on most workdays.	0.744
Sleep quality robustness outcome	T2	Item 4	My sleep quality was good during nonwork time.	0.740
Emotional exhaustion robustness outcome	T2	Item 1	I felt emotionally drained by my work.	0.757
Emotional exhaustion robustness outcome	T2	Item 2	I felt used up at the end of the workday.	0.761
Emotional exhaustion robustness outcome	T2	Item 3	I felt fatigued when thinking about another workday.	0.749
Emotional exhaustion robustness outcome	T2	Item 4	I felt burned out from my work demands.	0.757
Marker attention	T2	Item 1	I read survey items carefully.	0.753
Marker attention	T2	Item 2	I paid attention while answering the questionnaire.	0.765
Marker attention	T2	Item 3	I tried to answer consistently across the survey.	0.765
